# Intracellular Concentrations of *Borrelia burgdorferi* Cyclic Di-AMP Are Not Changed by Altered Expression of the CdaA Synthase

**DOI:** 10.1371/journal.pone.0125440

**Published:** 2015-04-23

**Authors:** Christina R. Savage, William K. Arnold, Alexandra Gjevre-Nail, Benjamin J. Koestler, Eric L. Bruger, Jeffrey R. Barker, Christopher M. Waters, Brian Stevenson

**Affiliations:** 1 Department of Microbiology, Immunology, and Molecular Genetics, University of Kentucky College of Medicine, Lexington, Kentucky, United States of America; 2 Department of Microbiology and Molecular Genetics, Michigan State University, East Lansing, Michigan, United States of America; 3 Department of Molecular Genetics and Microbiology, Center for Microbial Pathogenesis, Duke University, Durham, North Carolina, United States of America; Cornell University, UNITED STATES

## Abstract

The second messenger nucleotide cyclic diadenylate monophosphate (c-di-AMP) has been identified in several species of Gram positive bacteria and *Chlamydia trachomatis*. This molecule has been associated with bacterial cell division, cell wall biosynthesis and phosphate metabolism, and with induction of type I interferon responses by host cells. We demonstrate that *B*. *burgdorferi* produces a c-di-AMP synthase, which we designated CdaA. Both CdaA and c-di-AMP levels are very low in cultured *B*. *burgdorferi*, and no conditions were identified under which *cdaA* mRNA was differentially expressed. A mutant *B*. *burgdorferi* was produced that expresses high levels of CdaA, yet steady state borrelial c-di-AMP levels did not change, apparently due to degradation by the native DhhP phosphodiesterase. The function(s) of c-di-AMP in the Lyme disease spirochete remains enigmatic.

## Introduction

Several different compounds are produced by bacteria that serve as internal signals to control global gene expression and other functions. These include modified nucleotides such as cyclic-AMP and cyclic-di-GMP [1]. Fairly recently, a distinct cyclic molecule that consists of two AMP moieties, cyclic diadenylate monophosphate (c-di-AMP), was identified in some firmicute, actinomycete, and *Chlamydia* species [1–7]. This signaling molecule can significantly affect expression of numerous genes, and impact cell division, cell wall formation, and virulence [8–21]. In addition, bacterial c-di-AMP can invoke strong innate immune responses by eukaryotic hosts [2,5,22–26].

*Borrelia burgdorferi*, the Lyme disease spirochete, encounters numerous microenvironments during its vector-host infectious cycle. Efficient survival and transmission requires that the spirochete produces optimal levels of specific proteins and other components necessary for each step of the cycle. Upon sequencing the *B*. *burgdorferi* genome, it was surprising that this microbe encodes only two 2-component sensory/regulatory systems, two alternative sigma factors and very few other recognizable regulatory proteins [27]. However, in the intervening years, several previously-unknown types of regulatory proteins and messenger molecules have been discovered in Lyme disease spirochetes, and there may yet more to be uncovered [28,29]. Current understanding of *B*. *burgdorferi* regulatory pathways is far more complex than initially envisioned, with multiple interacting factors that cooperate or compete with each other to fine-tune borrelial protein expression patterns.

Herein, we describe that the *B*. *burgdorferi* genome contains a previously-unannotated open reading frame which encodes a protein with a “DAC” motif (di-adenlylate cyclase), a domain that contains conserved residues which are involved with synthesis of c-di-AMP. We now demonstrate that the encoded protein possesses the hypothesized enzymatic activity. As discussed in greater detail in the results section, the protein has been designated CdaA (cyclic di-AMP synthase), and that nomenclature will be used through the remainder of this report.

While this work was in progress, another research group also demonstrated that *B*. *burgdorferi* can produce c-di-AMP, although they did not identify the responsible enzyme [30]. Adding further significance to our characterization of *B*. *burgdorferi* c-di-AMP synthesis, those authors reported that the borrelial DhhP phosphodiesterase can degrade c-di-AMP. Inactivation of DhhP led to accumulation of c-di-AMP and altered expression levels of the alternative sigma factor RpoS and the virulence-associated OspC membrane protein [30].

We now show that, although expression of CdaA in the heterologous host *Escherichia coli* resulted in high level production of c-di-AMP, increased expression of CdaA in *B*. *burgdorferi* did not significantly impact the intracellular concentration of c-di-AMP. We conclude that changes to c-di-AMP levels in *B*. *burgdorferi* are not primarily driven by changing expression of CdaA.

## Materials and Methods

### In silico proteomic analyses

*B*. *burgdorferi* genome databases were analyzed by BLAST-P (http://www.ncbi.nlm.nih.gov/BLAST), restricting searches to the genus *Borrelia*. The *C*. *trachomatis* LGV-L2 c-di-AMP synthase (GenBank locus number YP_007715533) [5] was used as the query. Using Clustal X [31], the predicted sequence of *B*. *burgdorferi* CdaA was compared with sequences of other previously-defined c-di-AMP synthases: *Bacillus subtilis* CdaA (formerly YbbP, GenBank locus BAA19509), *Listeria monocytogenes* DacA (GenBank locus BN389_21520), *Staphylococcus aureus* (GenBank locus SAV2163), *C*. *trachomatis* DacA (GenBank locus YP_007715533).

Genomes of *Treponema* and *Leptospira* species were queried by BLAST-P using *B*. *burgdorferi* CdaA sequence as input, with output limited to those genera.

Sequenced *B*. *burgdorferi* genomes were also examined by BLAST-P for presence of homologs of the following c-di-AMP binding proteins that have been identified in other bacterial species: *M*. *smegmatis* DarR, GenBank locus ABK70852 [15]; *Streptococcus pneumoniae* CabP, GenBank locus SPD_0076 [16]; *Staphylococcus aureus* KtrA, GenBank locus SAUSA300_0988 [32]; *Staphylococcus aureus* CpaA, GenBank locus SAUSA300_0911 [32]; *Staphylococcus aureus* KdpD, GenBank locus AFH70306 [32]; and *Staphylococcus aureus* PstA, GenBank locus AFH69624 [32].

### Bacteria and plasmids

The *cdaA* gene was cloned from strain B31-MI-16, a derivative of the *B*. *burgdorferi* type strain [27,33]. Strain B31-e2, which lacks the wild-type restriction endonucleases, was used for all studies of transformed borreliae [34]. Control strain KS50 was derived from B31-e2 by transformation with the empty vector pSZW53-4 [35]. Borreliae were cultured in BSK-II broth at 35°C [36].

The *cdaA* open reading frame was PCR amplified using oligonucleotide primers CDAA-1 and CDAA-2 (Table 1). Primer CDAA-1 introduces a strong AGGAGG ribosome-binding site upstream of the *cdaA* initiation codon. The resultant amplicon was cloned in pCR2.1 (Invitrogen, Carlsbad, CA), and transformed into *E*. *coli* DH5α. The insert of the resultant plasmid was sequenced on both strands to confirm that mutations were not introduced during cloning methods, and that the *cdaA* ORF was oriented such that transcription could be driven by the vector’s *lac* promoter. This *E*. *coli* strain was designated CRS-0. Transcription of *cdaA* was induced in mid-exponential cultures of CRS-0 by addition of isopropyl-thiogalactoside (IPTG) to a final concentration of 60 μg/ml.

**Table 1:** Oligonucleotide primers used in these studies.

Name	Sequence (5’ to 3’)	Purpose
CDAA-1	TTGAGGAGGATCCTAATGATAGACATAAATG	Cloning *B*. *burgdorferi cdaA* for expression in *E*. *coli*
CDAA-2	TTCGGTACCTTACTCTATTAGCTCTAG	Cloning *B*. *burgdorferi cdaA* for expression in *E*. *coli*
CDAA-11	CCTATCAGTGATAGTGAAAAAGGAGGATCCTAATGATAGACATAAATG	PCR of *cdaA* for cloning into pSZW53-4
CDAA-12	CACAAGAGGCGACAGACTGCAGGTACCTTACTCTATTAGCTCTAG	PCR of *cdaA* for cloning into pSZW53-4
CDAA-13	CTAGAGCTAATAGAGTAAGGTACCTGCAGTCTGTCGCCTCTTGTG	PCR of pSZW53-4 for cloning *cdaA*
CDAA-14	CATTTATGTCTATCATTAGGATCCTCCTTTTTCACTATCACTGATAGG	PCR of pSZW53-4 for cloning *cdaA*
cdaA-F	CTCTTCACGATGGAGCTGTAAT	Q-RT-PCR analysis of *B*. *burgdorferi cdaA*
cdaA-R	GTCCTGCTCTATGTCTTGTTCC	Q-RT-PCR analysis of *B*. *burgdorferi cdaA*
qFlaB1	GGAGCAAACCAAGATGAAGC	Q-RT-PCR analysis of *B*. *burgdorferi flaB*
qFlaB2	TCCTGTTGAACACCCTCTTG	Q-RT-PCR analysis of *B*. *burgdorferi flaB*
recA-F	GCCGCTACAGAATCAACTACA	Q-RT-PCR analysis of *B*. *burgdorferi recA*
recA-R	GTTGCAGAACTTTGGCTTAGTC	Q-RT-PCR analysis of *B*. *burgdorferi recA*
ospC-F	CTTGCTGTGAAAGAGGTTGAAG	Q-RT-PCR analysis of *B*. *burgdorferi ospC*
ospC-R	CTCCCGCTAACAATGATCCA	Q-RT-PCR analysis of *B*. *burgdorferi ospC*
rpoS-F	TTTGGGACTATTGTCCAGGTTAT	Q-RT-PCR analysis of *B*. *burgdorferi rpoS*
rpoS-R	CCCTTGAACAAGATTCAACTCTAAA	Q-RT-PCR analysis of *B*. *burgdorferi rpoS*
rpoN-F	GGCCAATGAACTTGAGCATTT	Q-RT-PCR analysis of *B*. *burgdorferi rpoN*
rpoN-R	GCTCCACCAACAGAGCTAAA	Q-RT-PCR analysis of *B*. *burgdorferi rpoN*
bosR-F	TGCAATGCCCTGAGTAAATGA	Q-RT-PCR analysis of *B*. *burgdorferi bosR*
bosR-R	TGCAATCAAGTCCACCCTATTC	Q-RT-PCR analysis of *B*. *burgdorferi bosR*
csrA-F	ATGCTAGTATTGTCAAGAAA	Q-RT-PCR analysis of *B*. *burgdorferi csrA*
csrA-R	TGCTTATATTGTGTTTGTCT	Q-RT-PCR analysis of *B*. *burgdorferi csrA*
dhhP-F	CTTCTTCTAGCTCTGGCAAAGA	Q-RT-PCR analysis of *B*. *burgdorferi dhhP*
dhhP-R	CCCAACTATAATCGAACCATCCT	Q-RT-PCR analysis of *B*. *burgdorferi dhhP*

The *cdaA* ORF was then PCR amplified using primers CDAA-11 and CDAA-12 (Table 1). The *B*. *burgdorferi-E*. *coli* shuttle vector pSZW53-4 [35] was PCR amplified using primers CDAA-13 and CDAA-14. The two amplicons were annealed together by isothermal assembly [37], and *E*. *coli* DH5α was transformed with the assembly reaction mixture. The resultant plasmid, pAG1, was purified and the insert sequenced to confirm that no mutation had been introduced, and that the *cdaA* ORF was in the correct orientation. That construct was introduced into *B*. *burgdorferi* B31-e2, and transformant strain AG1 was selected by addition of kanamycin to 200 μg/ml [38].

For studies of the effects of *cdaA* hyperexpression, mid-exponential phase cultures (approximately 10^7^ bacteria/ml) of AG1 were equally divided into two tubes. Transcription of *cdaA* was induced by addition of 0.5 μg/ml (final concentration) anhydrotetracycline (ATc) to one tube, and both were incubated for 24h at 35°C. For each pair of induced/uninduced AG1 bacteria, equivalent aliquots were processed for total protein, RNA, and/or cytoplasmic extracts.

### SDS-polyacrylamide gel electrophoresis (SDS-PAGE) and immunoblotting

Bacterial protein contents were assessed by electrophoresis in SDS-PAGE and staining with Coomassie brilliant blue.

For immunoblot analyses, equal loading of *B*. *burgdorferi* cell extracts was assessed by immunoblot against the constitutively-expressed FlaB subunit of the flagella, using monoclonal antibody H9724 [39]. Rabbit polyclonal antisera directed against CdaA was obtained from NeoBioLab (Woburn, MA), who used as antigen a polypeptide consisting of CdaA residues 193–205, NVDSISKAFGTRH, using their standard protocol. Bound antibodies were detected using appropriate horseradish peroxidase-conjugated secondary antibodies and SuperSignal West Pico chemiluminescence reagent (Thermo Scientific).

### Analyses of c-di-AMP

*E*. *coli* lacks a native c-di-AMP synthetase, and is therefore a useful tool to determine whether or not a protein can produce c-di-AMP [5,9,13]. Thus, cytoplasmic extracts of IPTG-induced *E*. *coli* CRS-0 were produced to assess production of c-di-AMP by CdaA. Cytoplasmic extracts were also produced from induced and uninduced *B*. *burgdorferi* AG1. For all such analyses, equal volumes of cultures with equivalent concentrations of bacteria were harvested by centrifugation. Bacterial pellets were resuspended in equal volumes of extraction buffer (40:40:20 mixture of methanol, acetonitrile, and 0.1 N formic acid [by volume]), and incubated at -20°C for 30 min. Cellular debris was pelleted by centrifugation, supernatant decanted into a fresh tube, then stored at -80°C. c-di-AMP was quantified by ultra performance liquid chromatography—tandem mass spectrometry (UPLC-MS/MS) of equal volumes of each bacterial extract, as previously described [5,40].

### Quantitative reverse-transcription PCR (q-RT-PCR)

Total RNA was extracted from each set of induced and uninduced bacteria, and cDNA prepared according to previously described methods [41]. For each RNA sample, controls lacking reverse transcriptase were included to confirm absence of contaminating genomic DNA. Each strain and culture condition was independently replicated three times.

Oligonucleotide primer pairs were designed to specifically amplify the *B*. *burgdorferi cdaA*, *ospC*, *rpoS*, *rpoN*, *bosR*, *csrA*, *dhhP*, *flaB* and *recA* transcripts (Table 1). The specificity of each primer pair was tested by PCR of *B*. *burgdorferi* B31-MI total genomic DNA, and subsequent agarose gel electrophoresis and ethidium bromide staining. The borrelial *flaB* is generally considered to be constitutively expressed, and is commonly used as an internal standard against which expression levels of other transcripts are determined [41–44]. Ye et al. used an alternative internal standard, *recA*, for their analyses of the transcription effects of DhhP levels [30]. Both *flaB* and *recA* were used in the current study, in part to compare validity of the two targets as internal standards.

Levels of each target mRNA were assessed by Q-RT-PCR from each sample condition, and performed in duplicate. Transcript fold changes between uninduced and induced cultures of KS50 and AG1 were determined by the ΔΔCt method [45], using both *flaB* and *recA* as the standard. Multiple t tests between each transcript fold-changes were performed to determine significance, which were presented graphically (GraphPad Prism version 6.0 for Mac OS X, GraphPad Software, San Diego CA, www.graphpad.com).

## Results

### *B*. *burgdorferi* CdaA synthesizes c-di-AMP

**Fig 1:**
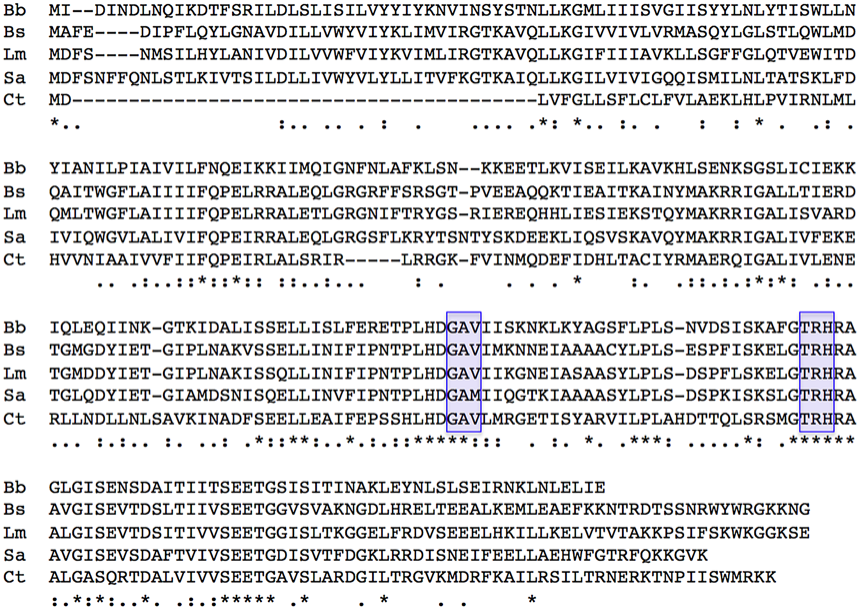
Alignment of the predicted amino acid sequences of *B*. *burgdorferi* CdaA and closely-related ci-di-AMP synthases of other bacteria. The two regions of conserved residues that constitute the DAC domain are boxed in blue. Residues found in all 5 proteins are indicated by an asterisk (*), residues in 4 proteins by a colon (:), and those in 3 proteins by a period (.). Enzyme sequences are identified as: Bb, *B*. *burgdorferi* CdaA; Bs, *Bacillus subtilis* CdaA (formerly YbbP); Lm, *Listeria monocytogenes* CdaA/DacA; Sa, *Staphylococcus aureus* DacA; and Ct, *C*. *trachomatis* DacA.

The GenBank bacterial genome database was analyzed by BLAST-P, using the *C*. *trachomatis* LGV-L2 c-di-AMP synthase as query. Only one potential homolog was identified in *B*. *burgdorferi* type strain B31, ORF BB0008, with an E value of 2 x 10^-30^. Significantly, the borrelial protein contains a consensus DAC domain (Fig 1). Alignment of the predicted borrelial gene product demonstrated extensive homology with other bacterial c-di-AMP synthases (Fig 1).

**Fig 2:**
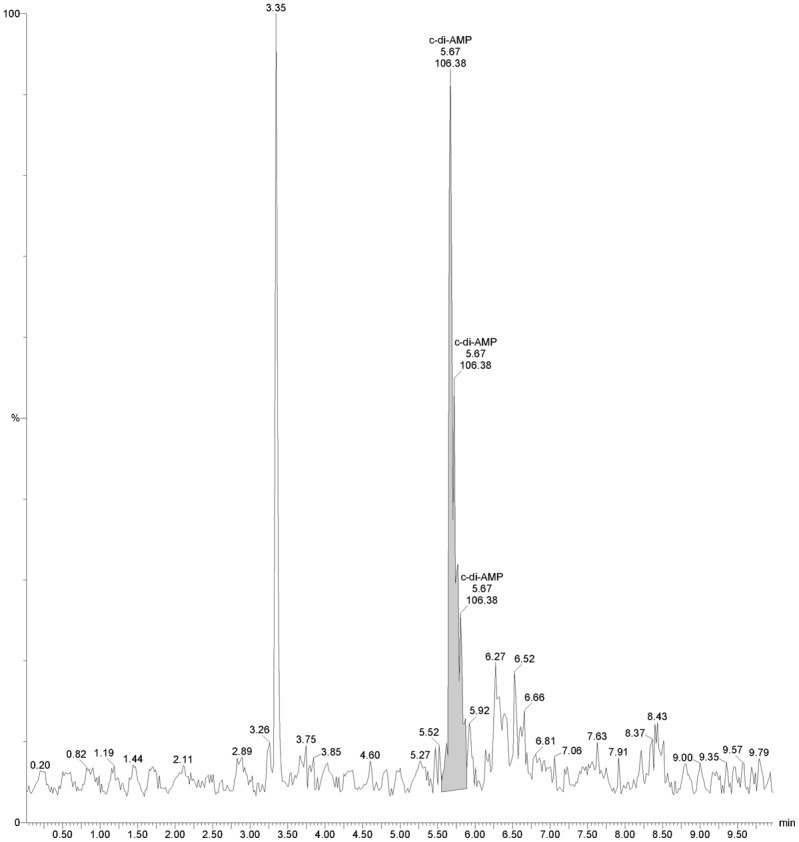
*B*. *burgdorferi* CdaA synthesizes c-di-AMP. Representative mass spectrometric analysis of cytoplasmic extract from IPTG-induced *E*. *coli* strain CRS-0, which expresses *B*. *burgdorferi* CdaA from a chimeric plasmid. The identity of the peak at 3.35 min was not determined.

*E*. *coli* does not naturally carry a gene for c-di-AMP synthase, so expression of an exogenous protein in *E*. *coli* is a simple means to determine that protein’s ability to produce c-di-AMP [5,9]. To that end, the identified borrelial ORF was cloned into *E*. *coli* vector pCR2.1, such that its transcription is directed by the vector’s *lac* promoter. The resultant plasmid was introduced into *E*. *coli* DH5α, producing strain CRS-0. Cytoplasmic extracts were prepared from induced CRS-0, then analyzed for presence of c-di-AMP by liquid chromatography coupled with tandem mass spectrometry. *E*. *coli* expressing the borrelial gene produced readily detectable levels of c-di-AMP (Fig 2).

Thus, it can be concluded that the *B*. *burgdorferi* gene encodes a c-di-AMP synthase. A recent proposal has been put forth that DAC domain proteins similar to the borrelial enzyme be named DacA [9]. However, that designation had long ago been given to bacterial D-alanyl-D-alanine carboxypeptidase [46], and *B*. *burgdorferi* possesses a gene for that enzyme (ORF BB0605) [27]. We decided not to unnecessarily confuse matters by giving the same name to two unrelated genes/proteins. Among the bacterial proteins with extensive similarities to the borrelial c-di-AMP synthase is the *Bacillus subtilis* CdaA (formerly YbbP) (Fig 1) [13]. A recent structural analysis of the *L*. *interrogans* c-di-AMP synthase also used the name CdaA [7]. We adopted that unambiguous name for the borrelial homolog.

### CdaA over-expression in *B*. *burgdorferi*

**Fig 3:**
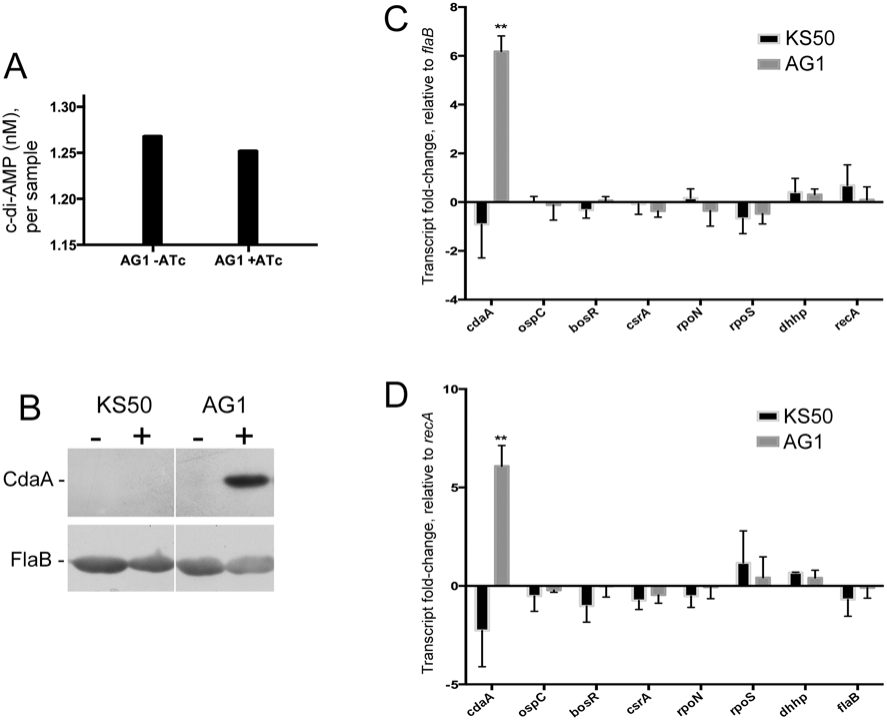
Effects of hyper-expressing CdaA in *B*. *burgdorferi*. **A.** Measurements of *B*. *burgdorferi* cytoplasmic c-di-AMP levels in samples of uninduced and induced AG1. Bacteria were cultured to mid-exponential phase (approximately 10^7^ bacteria/ml), divided equally divided into two tubes, then *cdaA* transcription was induced by addition of 0.5 μg/ml (final concentration) anhydrotetracycline (ATc) to one tube, and both were incubated for 24h at 35°C. Equal volumes of borrelial cell extracts were analyzed. **B.** Immunoblot analyses of KS50 and AG1, without and with inclusion of 0.5 μg/ml anhydrotetracycline (ATc) inducer (- and +, respectively). Membranes were probed with antibodies directed against CdaA or the constitutively-expressed FlaB subunit of the flagella. Wild-type and uninduced AG1 bacteria produced substantially less CdaA than did induced AG1, and the immunoblot signal was not detectable for those strains/conditions at the illustrated exposure. Analyses of mRNA levels also indicated that *cdaA* is expressed at low levels by uninduced AG1 (data not shown). **C and D.** Q-RT-PCR analyses of the effects of CdaA hyperexpression on transcription of select *B*. *burgdorferi* mRNAs. Transcript fold changes are shown as the difference between uninduced and induced cultures for both strains KS50 and AG1, relative to control *flaB* or *recA*, respectively [30]. Multiple t tests were performed for each strain and examined transcript. Only the differences in levels of *cdaA* transcripts in induced cultures of AG1 were significant (indicated by **, p = 0.0012 when compared with *flaB*, and p = 0.0023 when compared with *recA*).

Mass spectrometric analyses of wild-type *B*. *burgdorferi* cytoplasmic extracts indicated that cultured borreliae produce very low levels of c-di-AMP, which were barely above the threshold of detection (Fig 3A). Similarly low concentrations of cytoplasmic c-di-AMP were also observed by another research group [30]. Consistent with those observations, CdaA protein levels in cultured *B*. *burgdorferi* were found to be below the threshold of immunoblot detection (Fig 3B). Examination of published transcript array data of *B*. *burgdorferi* cultured under various conditions, or of regulatory mutants, failed to identify a condition or mutation that significantly altered *cdaA* expression [e.g., [47–52]]. Analyses of our published and unpublished data from RNA sequencing studies of additional *B*. *burgdorferi* mutants also did not identify significant regulation of *cdaA* expression [53] and unpublished results].

Production of c-di-AMP is essential for the survival of previously-studied bacterial species [13,14]. Noting also the low cellular levels of CdaA and c-di-AMP in cultured *B*. *burgdorferi* and our demonstration that increased production of CdaA in *E*. *coli* resulted in high-level synthesis of c-di-AMP, we examined the effects of hyperexpression of CdaA on *B*. *burgdorferi*. Depletion of the DhhP phosphodiesterase blocks borrelial growth [30], so we avoided use of a *dhhP* mutant for these studies. To that end, strain AG1 was produced, in which *cdaA* transcription is under control of the TetR-regulated hybrid P*ost* promoter [35,54]. Q-RT-PCR analysis indicated that induction of *cdaA* in AG1 increased its mRNA levels by 6-fold, and immunoblot analysis confirmed greatly enhanced production of the CdaA protein (Fig 3B, 3C and 3D). However, analyses of cytoplasmic extracts from induced AG1 indicated wild-type levels of c-di-AMP (Fig 3A). The insert of the *cdaA*-expression plasmid was purified from AG1, re-sequenced, and found to be identical to the native *cdaA* gene, indicating that the continued low levels of c-di-AMP were not due to a mutation in the introduced enzyme. Hyperexpression of CdaA did not produce any detectable effects of borrelial growth rate, cell size or survival (data not shown). There were also no significant effects on mRNA levels of *dhhP*, *ospC* or the regulatory factors *rpoS*, *rpoN*, *bosR* or *csrA* (Fig 3C and 3D).

## Discussion

Bacterial production of c-di-AMP has been detected in some firmicute species, the actinomycetes *Mycobacterium tuberculosis* and *smegmatis*, the chlamydian *C*. *trachomatis*, and the spirochete *B*. *burgdorferi*. DAC motif-containing CdaA homologs are found throughout the spirochete phylum, including the syphilis agent *Treponema pallidum* and other members of that genus (e.g., *T*. *pallidum* Nichols ORF TP0826), and *Leptospira interrogans* and other leptospires (e.g., *L*. *interrogans* Copenhageni ORF LIC10844 and *L*. *biflexa* Patoc 1 ORF LEPBI_I0735) [55–57]. It is not obvious why production of this modified nucleotide is restricted to only a few phyla, but absent from proteobacteria and so many others [6].

Since expression of CdaA in *E*. *coli* led to significant accumulation of c-di-AMP by that bacterium, we hypothesized that enhanced CdaA levels in *B*. *burgdorferi* would similarly lead to increased c-di-AMP production. However, increased levels of the CdaA enzyme in *B*. *burgdorferi* did not measurably affect steady-state cytoplasmic c-di-AMP levels. In contrast, depletion of the *B*. *burgdorferi* DhhP phosphodiesterase led to increased cytoplasmic levels of c-di-AMP [30]. Those data suggest that DhhP and/or some other enzymatic activity is responsible for maintaining the constant, low levels of c-di-AMP in both wild-type and induced AG1 borreliae.

The results of these studies and those of Ye et al. [30] raise an important question about the function of c-di-AMP in *B*. *burgdorferi*: why is this molecule, which uses up 2 ATP molecules, produced by CdaA but then destroyed? To date, no signal has been identified that affects expression levels of CdaA. *B*. *burgdorferi* does control expression of *dhhP* [30]. However, conditional depletion of DhhP led to an approximately 40-fold increase in c-di-AMP concentration, along with a cessation of growth, while ectopic modulation of DhhP that yielded a 5-fold increase in c-di-AMP levels did not have any noticeable effects on growth or cell division [30]. Thus, there is an apparently broad window of c-di-AMP levels that can be tolerated by *B*. *burgdorferi* without having a detectable impact on the bacteria. Whether c-di-AMP directly controls *B*. *burgdorferi* growth, division, and/or regulatory factors remains to be determined, since the observed phenotypes may be indirect responses to stresses induced by disruption of another bacterial function(s). It is also possible that the DhhP phosphodiesterase acts on substrates other than c-di-AMP, which may be responsible for the growth defects when DhhP is depleted.

The field of bacterial c-di-AMP signaling is still in its infancy, and is not well understood in any species. Of the c-di-AMP-binding proteins that have been identified in other bacteria, homologs of the following are present in *B*. *burgdorferi*: ORF BB0724 is orthologous to *Streptococcus pneumoniae* CabP (E = 6x10^-27^), BB0725 to *Staphylococcus aureus* KtrA (E = 7x10^-21^), and both ORFs BB0216 and BB217 to *Staphylococcus aureus* PstA (E = 7x10^-21^, and E = 7x10^-21^, respectively) [16,27,32,58–60]. Those streptococcal and staphylococcal proteins are all involved with potassium transport, so the similarities with borrelial proteins may simply be due to that function. Nonetheless, examination of interactions between c-di-AMP and *B*. *burgdorferi* phosphate transport proteins, and the significance of any such binding, may be a fruitful venue for future studies.

Riboswitches dependent upon c-di-AMP have been identified in some bacterial species, which may affect gene expression [61–64]. To the best of our knowledge, the possibility of riboswitches being present in *B*. *burgdorferi* has yet to be explored. The oral spirochete *Treponema denticola* contains a thymidine pyrophosphate-dependent riboswitch [65], suggesting that such regulatory mechanisms may exist in other spirochetes.

Another potential role for CdaA and DhhP is production and degradation of di-AMP (pApA), which is the initial c-di-AMP breakdown product. That dinucleotide may serve as a nanoRNA, which could have wide-ranging impacts upon transcription initiation [66–68]. We note also that many different nanoRNAs are produced and degraded in other bacterial species by DHH-motif enzymes, supporting the possibility that *B*. *burgdorferi* DhhP might degrade a broader variety of nucleic acids than just c-di-AMP [66–71].

Borrelial c-di-AMP may have impacts beyond the bacterium itself. c-di-AMP produced by *L*. *monocytogenes* and *C*. *trachomatis* activates a type I interferon response by host cells [2,5,22–25]. Although those bacteria invade host cells, while *B*. *burgdorferi* is an extracellular pathogen, it is possible that a portion of the observed type I interferon responses observed during *B*. *burgdorferi* infection might be linked to the spirochete’s c-di-AMP [29,72–74].

In summation, these studies demonstrated that *B*. *burgdorferi* produces an enzyme, CdaA, that synthesizes c-di-AMP. Homologs of CdaA are found throughout the spirochete phylum. We hypothesize that this modified nucleotide is rapidly broken down by the DhhP phosphodiesterase. Thus, regulation of CdaA did not significantly affect cytoplasmic levels of c-di-AMP, and we predict that other mechanisms, such as factors that control the activity of CdaA or altered expression of DhhP, are the major drivers of altering c-di-AMP levels.
